# Comparative Analysis of the Structural and Digestibility Properties of Starches from Ten Common Coarse Grains

**DOI:** 10.3390/foods15172946

**Published:** 2026-08-22

**Authors:** Zulipiya Maimaiti, Hong-Yan Mao, Hong-Nan Sun, Li Yue, Jiamin Wang, Tingting Zhang, Yueren Xu, Ming Yu, Tai-Hua Mu

**Affiliations:** 1 Institute of Crop Research, Xinjiang Academy of Agricultural Sciences, Urumqi 830091, China; zulipiya83@126.com (Z.M.); maohongyan1226@126.com (H.-Y.M.); yueli4467@163.com (L.Y.); xiao_xin227@163.com (J.W.); ztt930615@163.com (T.Z.); 15136705752@163.com (Y.X.); 2 Institute of Food Science and Technology, Chinese Academy of Agricultural Sciences, Beijing 100193, China; sunhongnan@caas.cn

**Keywords:** coarse grains, starches, structural analysis, rheological properties, in vitro starch digestibility

## Abstract

Coarse-grain starches are promising raw materials for developing diversified functional food ingredients, particularly low-glycemic products. However, the systematic structure–function relationships among multiple coarse-grain varieties remain poorly understood. This study aimed to comprehensively characterize the structural, processing, and digestive properties of ten coarse-grain starches to provide a fundamental basis for their targeted industrial utilization. Multiple analytical techniques, including X-ray diffraction (XRD), Fourier-transform infrared spectroscopy (FT-IR), differential scanning calorimetry (DSC), rapid visco analysis (RVA), and in vitro simulated digestion, were employed to characterize the structural, thermal, rheological, pasting, and digestive properties of the starches. The tested starches were classified into two crystalline types, and significant differences were observed among samples in short-range molecular order, gelatinization behavior, gel rheological properties, pasting characteristics, and the distribution of three digestion fractions. Pearson correlation analysis revealed structure–function correlations, showing that crystalline ordering plays an important role in starch gelatinization behavior, whereas molecular packing is closely associated with pasting performance and in vitro digestibility. The distinct differences in starch properties, together with the identified structure–function relationships, provide a basis for targeted raw material selection in food processing and the development of functional foods with tailored digestibility characteristics.

## 1. Introduction

Coarse grains, a diverse group of crops including cereals (e.g., millet, sorghum, oats, and maize), pseudocereals (e.g., buckwheat and quinoa), and pulses (e.g., mung beans, chickpeas, and red beans), are broadly defined as grains other than wheat and rice [1]. These crops are of global importance, particularly in arid and semi-arid regions, where they serve as staple foods and important sources of essential nutrients, contributing to food security and dietary diversity [2].

Starch, the primary component of these grains, typically accounting for 37% to 80% of their dry weight, is a key determinant of their nutritional and functional properties. In recent years, starches derived from these underutilized crops have attracted growing research interest due to their distinctive physicochemical characteristics. For instance, legume starches generally exhibit higher gelatinization temperatures, higher resistant starch and amylose contents, greater shear-thinning behavior, and lower digestibility than conventional cereal starches [3,4].

In contrast, starches from cereal and pseudocereal coarse grains, such as millet, tartary buckwheat, and quinoa, exhibit distinct physicochemical characteristics. Millet starches typically have granule sizes ranging from 0.3 to 24 μm and amylose contents ranging from 6% to 38.6%, with predominantly polygonal granules [5]. Tartary buckwheat starch is characterized by irregular polygonal granules with an average particle size of approximately 6.8 μm, and its paste exhibits strong hot- and cold-paste stability and good gel-forming ability [6]. Quinoa starch is notable for its exceptionally small granule size (1 to 3 μm) and unique freeze–thaw stability, making it suitable for frozen foods and other specialized applications [7]. These distinctive properties provide important processing advantages, making these starches suitable for use as thickening agents in noodles and vermicelli, stabilizers in canned foods, fat replacers in meat products, and texture enhancers in baked goods [8,9,10]. Structural differences among starches further determine their specific applications. For example, the favorable amylose sedimentation properties of mung bean and pea starches make them well suited for vermicelli production [11]. The highly branched structure and high molecular weight of red bean starch facilitate its use in puffed snacks and pastes [12]. Similarly, the high viscosity and low water absorption of fava bean starch make it suitable for pastries and bread [13].

Despite the diverse applications of legume, cereal, and pseudocereal coarse grain starches, a comprehensive understanding of their structure–function relationships remains limited. Previous comparative studies have primarily focused on individual starch sources, with relatively few systematic comparisons across different botanical origins. In addition, direct comparisons among studies are often complicated by differences in analytical methods and experimental conditions. To address this gap, ten commonly consumed coarse-grain varieties representing three botanical categories, cereals (millet, oat germ rice, and maize), pseudocereals (tartary buckwheat and white quinoa), and pulses (mung bean, red kidney bean, chickpea, red bean, and black kidney bean), were selected to enable a comprehensive and representative comparison. This design enables the systematic evaluation of structural and functional diversity both within and across botanical categories. Moreover, correlations across multiple structural levels, ranging from molecular order and crystalline organization to granular morphology, and their collective influence on functional properties such as pasting behavior, gelation, and digestibility, have not been systematically evaluated for such a broad range of coarse-grain starches under standardized analytical conditions. This integrated, multi-technique approach provides a unique opportunity to identify both category-specific and universal structure–function relationships, thereby supporting the rational selection of starches for targeted functional food applications.

## 2. Materials and Methods

### 2.1. Materials

**Figure 1:**
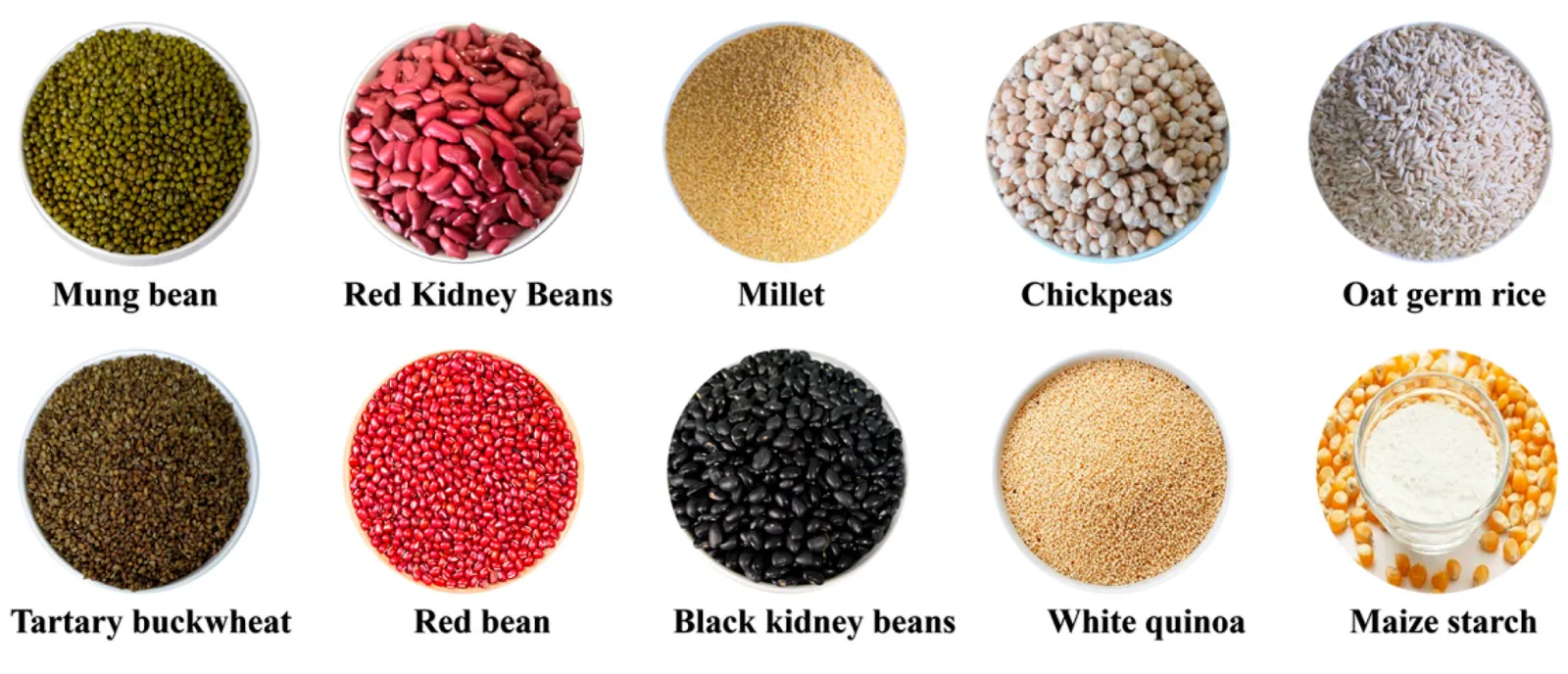
Photographs of seeds from ten common coarse grains.

Seeds of mung bean (*Vigna radiata*, MB), red kidney bean (*Phaseolus vulgaris*, RK), millet (*Setaria italica*, Mi), chickpea (*Cicer arietinum*, Ch), oat germ rice (*Avena sativa*, OG), tartary buckwheat (*Fagopyrum tataricum*, TB), red bean (*Vigna angularis*, RB), black kidney bean (*Phaseolus vulgaris*, BK), white quinoa (*Chenopodium quinoa*, WQ), and maize (*Zea mays*, Ma) were used in this study (Figure 1). Commercial maize starch (*Zea mays*, Ma) was purchased from a local supplier. All starches, except commercial maize starch, were extracted from the corresponding grains according to the procedure described in Section 2.2.

The coarse-grain seeds were purchased from Jianghe Market in Urumqi, China, in 2023. However, specific cultivar information and crop year were unavailable because the raw materials were commercially sourced, representing a limitation of this study. The seeds were stored at room temperature (25 ± 2 °C) in airtight containers under dry, dark conditions before starch extraction.

Porcine pancreatic α-amylase (P7545, 8 U/mg) was purchased from Sigma-Aldrich (Shanghai, China). Amyloglucosidase (S10017, 100,000 U/g) was purchased from Shanghai Yuanye Bio-Technology Co., Ltd. (Shanghai, China). All other chemical reagents were of analytical grade.

### 2.2. Starch Extraction

Starch was extracted using wet grinding and sedimentation techniques, following the methods described by Zeng Feng et al. [14] and Gao Jinfeng et al. [15], with minor modifications. Briefly, grain samples, including oat germ rice, tartary buckwheat, millet, and quinoa, were crushed and passed through an 80-mesh sieve. Legumes, including mung beans, red beans, chickpeas, red kidney beans, and black kidney beans, were soaked in sodium hydroxide (NaOH) solution to loosen the seed coats, then dehulled, pulped, and ground. The use of NaOH for legume dehulling is a common pretreatment method that facilitates seed coat removal and improves starch recovery. In contrast, cereals and pseudocereals do not require this step because of their thinner hulls and distinct seed structures [14,15]. Following these initial treatments, all samples underwent identical subsequent processing steps, including filtration, washing, ethanol treatment, and drying, to ensure comparability among extracted starches. The alkaline soaking solution was prepared at a 1:10 sample-to-solution ratio using 0.1% NaOH and shaken at 40 °C for 2 h. The resulting mixture was filtered through a 200-mesh sieve, allowed to settle, and washed thoroughly with distilled water. The collected starch was then washed with 80% ethanol until the supernatant became clear and free of pigments. Finally, the purified starch was dried at 50 °C for 48 h to obtain the final product.

### 2.3. Color Analysis

The color parameters of raw and processed samples were measured using a color difference meter (CR-400, Konica Minolta, Tokyo, Japan). A standard white calibration tile was used to calibrate the instrument before measurements. Color parameters were expressed in the CIE L*a*b* color space, where L* represents lightness (100 = white, 0 = black), a* represents the red–green axis (positive values indicate red, negative values indicate green), and b* represents the yellow–blue axis (positive values indicate yellow, negative values indicate blue). Additionally, the color intensity or saturation (chroma, C), hue angle (H°), and total color difference (ΔE) were calculated using the following formulas:

(1) C = (a*^2^ + b*^2^)^1/2^

(2) H° = tan^−1^ (b*/a*)

(3) ΔE = [(L* − L*_r_) ^2^ + (a* − a*_r_) ^2^ + (b* − b*_r_) ^2^]^1/2^

where L*, a*, and b* represent the color parameters of the starch sample; and L*r, a*r, and b*r represent those of the reference standard (white calibration tile).

### 2.4. Amylose Content, Moisture Content, and Ash Content Analysis

The apparent amylose content of the extracted starches was determined using an amylose content assay kit (Solarbio, Beijing, China; Cat. No. BC4265) based on the iodine colorimetric method. Briefly, starch samples (approximately 10 mg) were defatted with 85% ethanol to remove soluble sugars and lipids. The defatted starch was then dispersed in 1 M NaOH and heated in a boiling water bath for 10 min to ensure complete gelatinization. After cooling to room temperature, the solution was neutralized with acetic acid and diluted to an appropriate concentration. An aliquot of the solution was reacted with iodine reagent (I_2_-KI solution) to form a blue amylose-iodine complex. The absorbance was measured at 550 nm and 485 nm using a microplate reader. Amylose content was calculated using a standard curve generated with a pure amylose standard (Sigma-Aldrich). All measurements were performed in triplicate.

Moisture content was determined using a rapid moisture analyzer at 105 °C until a constant weight was achieved.

Ash content was determined according to the national standard method GB 5009.4-2016 [16]. Briefly, starch samples (2.00 g) were placed in pre-weighed crucibles, charred on a hot plate, and incinerated in a muffle furnace at 550 °C for 4 to 6 h until white or light gray ash formed. The residue was cooled in a desiccator and weighed. All measurements were performed in triplicate.

### 2.5. Scanning Electron Microscopy (SEM)

The micromorphology of coarse-grain starch granules was examined using a scanning electron microscope (SEM) at a magnification of 500×, an accelerating voltage of 2.0 kV, a working distance of 6.9 mm, and a secondary electron detector (SE2). Before observation, the raw starch samples (dried at 35 °C) were mounted onto aluminum stubs using double-sided carbon adhesive tape and coated with a thin layer of gold using a sputter coater under vacuum conditions following the standard procedure of the testing center. Representative images were selected from multiple regions of each sample to ensure the reproducibility of morphological characteristics.

### 2.6. Fourier Transform Infrared (FT-IR) Spectroscopy

The FT-IR spectra of starch samples were recorded using a spectrometer (INVENIO, Bruker, Germany) at room temperature. Dried starch powder (1 mg) was mixed with 100 mg potassium bromide (KBr) and pressed into pellets with a thickness of 1 to 2 mm. FT-IR spectra were collected over a wavenumber range of 4000–400 cm^−1^ at a resolution of 4 cm^−1^ with 64 scans per spectrum [17]. The spectra were baseline-corrected and normalized over the 1200 to 800 cm^−1^ spectral range. The ratio of absorbance at 1047 cm^−1^ to 1022 cm^−1^ (R_1047/1022_) was calculated to evaluate the short-range molecular order of the starch granules [18].

### 2.7. X-Ray Diffraction (XRD) Analysis

The crystalline structure of the starch samples was analyzed using an X-ray diffractometer (D8 Advance, Bruker, Germany) equipped with Cu Kα1 radiation (λ = 1.5418 Å) and operated at 40 kV and 40 mA. Diffraction patterns were recorded in locked-coupled scan mode over a 2θ range of 5° to 40°, with a step size of 0.02°, and a scan rate of 2°/min. Relative crystallinity was calculated as the ratio of the crystalline peak area to the total diffraction area using Origin software (OriginLab, Northampton, MA, USA). An amorphous baseline was drawn between the diffraction minima for background subtraction. All measurements were performed in triplicate.

### 2.8. Differential Scanning Calorimetry (DSC)

Approximately 3 mg of starch was mixed with 6 μL of distilled water and thoroughly homogenized. The mixture was then sealed in an aluminum pan and equilibrated at room temperature for 2 h. The sample was heated from room temperature to 110 °C at a rate of 10 °C/min for thermal analysis using a differential scanning calorimeter (DSC3500, NETZSCH, Selb, Germany) [15].

### 2.9. Rapid Visco Analyzer (RVA)

The pasting properties of the starches were determined using a Rapid Visco Analyzer (RVA-4500, Perten Instruments Newport Scientific Pty Ltd., Sydney, Australia). Starch suspensions were prepared at 10% (*w*/*w*, dry starch basis). The instrument software automatically calculated the required sample mass based on the measured moisture content to ensure a constant dry starch mass (approximately 2.5 g of dry starch in 25.0 mL of distilled water). The starch suspension was transferred into the RVA sample cell and analyzed using the standard temperature program at a rotation speed of 160 rpm: equilibration at 50 °C for 1 min, heating to 95 °C at 12 °C/min, holding at 95 °C for 2.5 min, cooling to 50 °C at 12 °C/min, and holding at 50 °C for 2 min. Pasting properties were evaluated based on peak viscosity (PV), trough viscosity (TV), breakdown viscosity (BDV), final viscosity (FV), setback viscosity (SV), peak time (PeT), and pasting temperature (PaT). All measurements were performed in triplicate.

### 2.10. Rheological Properties

Steady and dynamic shear rheological properties were determined following a previously described method [19]. Measurements were performed using a rheometer (MCR102e, Anton Paar, Graz, Austria) equipped with a parallel-plate geometry (diameter: 40 mm, gap: 1 mm) at 25 °C. The starch pastes used for rheological measurements were prepared as described in Section 2.9 using 10% (*w*/*w*) starch suspensions subjected to the standard RVA heating and cooling program. After completion of the RVA, the resulting starch pastes were immediately transferred to the rheometer platform for measurement. Steady shear rheological properties were measured at shear rates ranging from 0 to 100 s^−1^ at 25 °C. For dynamic rheological measurements, frequency sweep tests were performed at 25 °C with a strain amplitude of 1%, which was within the linear viscoelastic region, as described previously [20]. Dynamic rheological properties, on the other hand, were assessed over an angular frequency range of 0.1–100 rad/s [20]. The experimental data were fitted using the Herschel–Bulkley model [21], which is widely applied to describe the flow behavior of non-Newtonian fluids in both theoretical and practical applications, using the following equation:

Herschel–Bulkley model:

(4) σ = σ_0_ + Kγ^n^

where σ represents the shear stress (Pa), σ_0_ represents the yield stress (Pa), γ represents the shear rate (s^−1^), K represents the consistency coefficient (Pa.S^n^), and n represents the flow behavior index. The coefficient of determination (R^2^) represents the goodness of fit for the model.

### 2.11. Texture Profile Analysis (TPA) of Starch Gels

The textural properties of gelatinized starch gels were evaluated using a texture analyzer (TMS-Pilot, Bosin, Shanghai, China) after RVA. To promote retrogradation and gel formation, samples were stored in metal canisters at 4 °C for 24 h, resulting in cylindrical gels approximately 30 mm in diameter and 15 mm in height. A cylindrical aluminum probe (P/36R, 36 mm diameter) was used to compress the gels at 1 mm/s to 30% of their original height, with a 1 s interval between the two compression cycles. The collected data were analyzed to determine hardness, cohesiveness, springiness, adhesiveness, chewiness, and stickiness of the gels [22]. In this study, adhesiveness was defined as the work (force × distance) required to overcome the attractive forces between the gel and the probe surface during probe withdrawal, represented by the negative area under the force-time curve. In contrast, stickiness was defined as the maximum negative force (peak force) required to separate the probe from the gel surface during the withdrawal phase [22].

### 2.12. In Vitro Starch Digestibility

The kinetics of starch digestion were determined following a previously described method [23], with minor modifications. Raw starch samples (150 mg, dry weight basis) were suspended in 9 mL of acetate buffer (0.2 M, pH 6.0, containing 200 mM CaCl_2_ and 0.5 mM MgCl_2_) and equilibrated at 37 °C. Subsequently, 1 mL of mixed enzyme solution containing 10 U/mL α-amylase (P7545, Sigma-Aldrich, 8 U/mg) and 18 U/mL amyloglucosidase (S10017, Shanghai Yuanye, Shanghai, China, 100,000 U/g) was added. The enzyme solutions were freshly prepared by dissolving the required amounts of enzyme powder in acetate buffer to achieve final concentrations of 10 U/mL α-amylase and 18 U/mL amyloglucosidase, corresponding to approximately 1.25 mg/mL α-amylase and 0.18 mg/mL amyloglucosidase, respectively. Aliquots (120 μL) of the enzymatic hydrolysate were collected at 0, 10, 20, 30, 60, 120, 150, and 180 min and immediately mixed with 120 μL of 95% ethanol (*v*/*v*) to inactivate the enzymes. The remaining starch residue was separated by centrifugation at 2000× *g* for 5 min, and glucose concentration was determined using the GOPOD assay. The starch fractions were calculated as rapidly digestible starch (RDS) (hydrolyzed portion within 0 to 20 min), slowly digestible starch (SDS) (hydrolyzed portion within 20 to 120 min), and resistant starch (RS) (undigested portion after 120 min) using the following equations [24]:

(5) RDS (%) = [(G20 − G0) × 0.9/TS] × 100

(6) SDS (%) = [(G120 − G20) × 0.9/TS] × 100

(7) RS (%) = [100 − RDS − SDS]

where G represents the glucose concentration (g/100 g dry weight), and TS represents the total starch content (g/100 g dry weight). TS was measured using a total starch kit (K-TSTA-100A, Megazyme International, Wicklow, Ireland).

### 2.13. Kinetics of Starch Hydrolysis

The kinetics of starch digestion were analyzed using a first-order equation introduced by Guggenheim to estimate the rate constant of first-order reactions [25]:

(8) C_i+1_ − C_i_ = C_∞_e^−kt^(1 − e^−kΔ^),

where C_∞_ represents the concentration of digested starch at the reaction endpoint, C represents the concentration measured at time points separated by a constant interval Δt (i.e., the intervals between t1, t2, t3, etc., are all Δt), and k represents the pseudo-first-order rate constant.

### 2.14. Statistical Analysis

Analysis of variance (ANOVA) was performed to evaluate differences among means, followed by Duncan’s multiple range test at a significance level of *p* < 0.05. Pearson’s correlation analysis was used to assess the correlation between variables. Data analyses were performed using SPSS version 20.0 (SPSS Inc., Chicago, IL, USA). Experiments were performed in triplicate, and results were reported as mean ± standard deviation.

## 3. Results and Discussion

### 3.1. Color

Color is a crucial factor in evaluating starch quality, as it directly affects consumer acceptance and industrial applicability [26,27]. Significant differences (*p* < 0.05) were observed among the coarse-grain starches in color parameters, including L* (lightness), a* (red–green axis), b* (yellow–blue axis), and whiteness index (Table 1). Ma and OG starches exhibited the highest L* values, at 96.57 and 96.17, respectively, whereas RB starch had the lowest value at 88.75. The a* values, which represent redness (positive values) and greenness (negative values), ranged from −1.84 (MB and BK starches) to 1.00 (RB starch). Notably, RB starch exhibited a significantly higher a* value (*p* < 0.05) than the others, indicating a stronger reddish hue. The b* values, which represent yellowness when positive, ranged from 1.43 (OG starch) to 11.11 (RB starch). Compared with the other coarse-grain starches and maize starch, OG starch exhibited a relatively lower b* value, indicating less yellowness. The whiteness index of the starches ranged from 84.16 to 98.45, with OG, WQ, Ch, and Mi starches exhibiting significantly higher whiteness values, while RB starch had the lowest (*p* < 0.05).

Overall, the color characteristics of most coarse-grain starches were comparable to those of maize starch. The distinctive reddish color of RB starch (a* = 1.00, b* = 11.11, W = 84.16) is likely attributable to the presence of anthocyanin pigments in the red bean seed coat [26]. These pigments may have been released during alkaline soaking (0.1% NaOH) and subsequent pulping steps and may have interacted with starch granules through hydrogen bonding or electrostatic interactions [27]. Although the extraction protocol included washing with 80% ethanol to remove pigments, some anthocyanins may not have been completely removed due to their strong interactions with starch granules, resulting in the observed reddish hue. Similar observations have been reported for other pigmented legume starches [28].

The functional properties of starches are influenced not only by their molecular structure but also by residual non-starch components, particularly proteins and lipids [29]. In this study, the apparent amylose content of the ten starches ranged from 3.34% (WQ) to 21.81% (OG), with legume starches generally exhibiting higher amylose contents (13.86% to 20.89%) than most cereal and pseudocereal starches (3.34% to 21.81%). These values are generally consistent with those reported previously for the corresponding starch types.

Although color parameters, particularly L* and the whiteness index, can serve as indirect indicators of starch purity, they do not replace direct chemical analysis of residual non-starch components. The high L* values (>88) observed for all starches suggest that the extraction procedure effectively removed most pigmented impurities. However, because protein and lipid contents were not quantified due to sample limitations, their potential influence on the measured functional properties, including pasting viscosity, gel texture, and digestibility, cannot be entirely excluded. Future studies should include proximate composition analysis to further clarify the relative contributions of starch structure and residual non-starch components to the observed functional properties.

### 3.2. SEM

**Figure 2:**
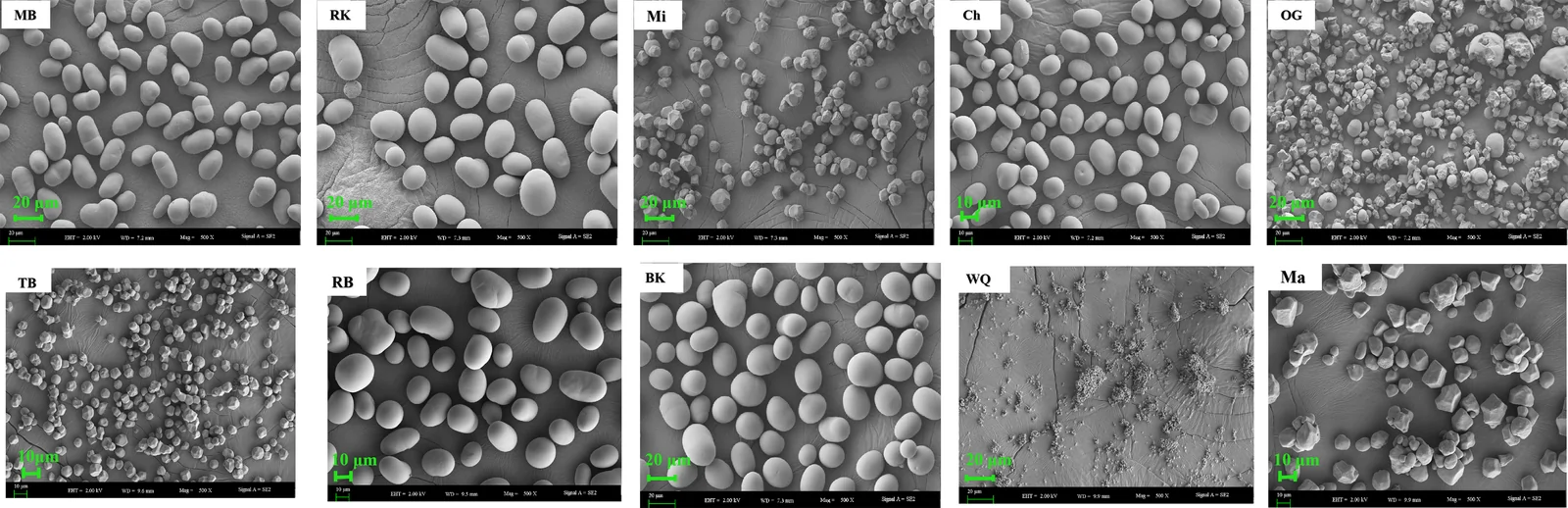
Scanning electron micrographs of starch granules from ten common coarse grains (500× magnification).

Figure 2 illustrates the morphological and surface characteristics of coarse-grain starch granules. Starch granules from MB, RK, Ch, RB, and BK predominantly exhibit spherical or elliptical shapes with smooth, crack-free surfaces and considerable variation in granule size, with both large and small granules present. These morphological features are consistent with previous reports on legume starches, which commonly exhibit oval, elliptical, or kidney-shaped granules with smooth surfaces [11,30,31]. In contrast, starch granules from Mi, OG, TB, WQ, and Ma exhibited irregular polygonal shapes, which are typical of cereal and pseudocereal starches [32,33,34]. These granules appeared smaller and more uniform in size than those of legume starches. The apparent aggregation of some granules may be partially attributed to drying during sample preparation. These morphological differences may result from inherent variations in the botanical characteristics of coarse grains, which influence the biosynthetic pathways governing starch granule formation and organization.

### 3.3. FT-IR

**Figure 3:**
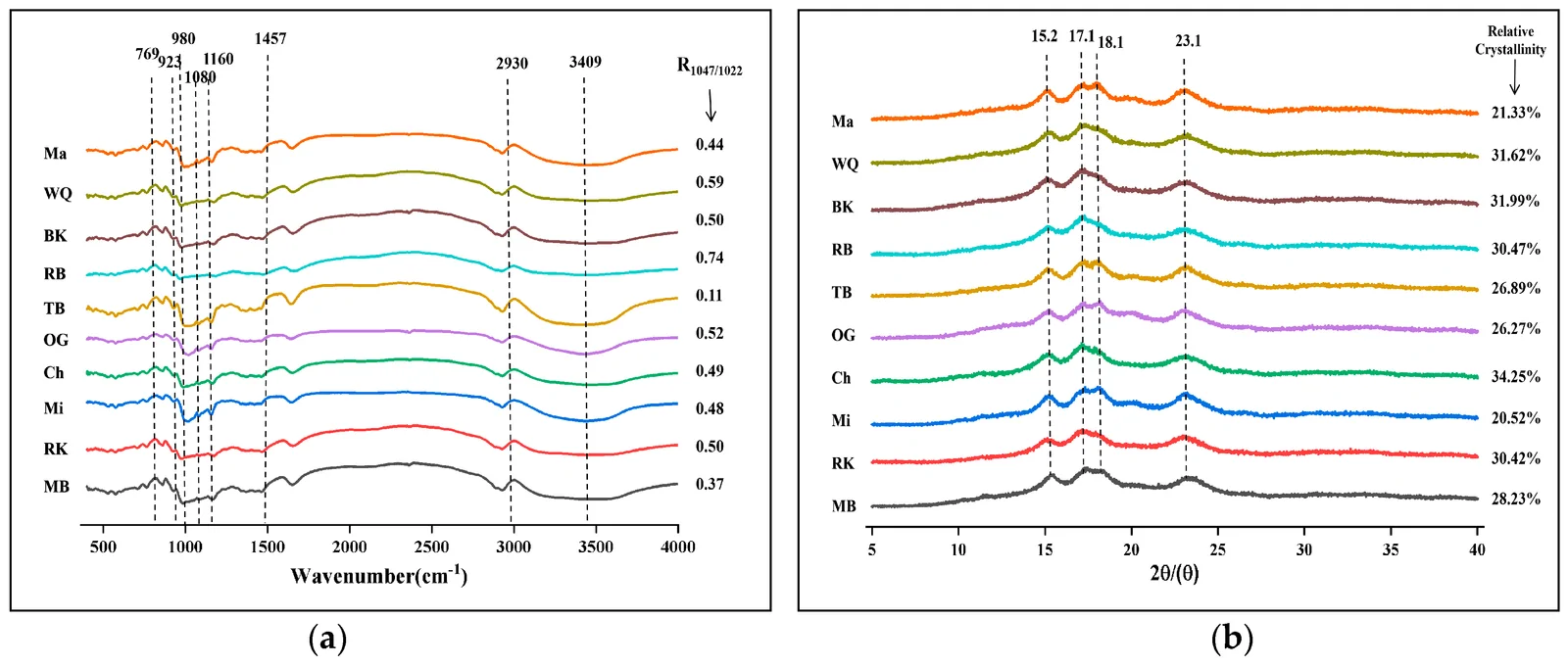
FTIR spectra (**a**) and X-ray diffraction patterns (**b**) of starches from ten common coarse grains.

The short-range molecular order of the ten coarse-grain starches was characterized by FT-IR spectroscopy based on characteristic infrared absorption bands. As shown in Figure 3a, distinct and sharp absorption peaks were observed at 3409, 2930, 1160, 1457, 1080, 980, 923, and 769 cm^−1^. These bands corresponded to the bending and stretching vibrations of hydroxyl groups involved in intermolecular hydrogen bonding within starch polysaccharides. A characteristic C-H stretching vibration was detected at 2930 cm^−1^, while the broad, well-defined band around 3409 cm^−1^ indicated the stretching vibration of hydrogen-bonded O-H groups [18].

The absorption peak at 1457 cm^−1^ was attributed to -CH bending vibrations, whereas the peaks at 1160, 1080, and 980 cm^−1^ corresponded to C-O-O stretching vibrations associated with the glucopyranose ring or glycosidic bonds in starch molecules [18]. The sharp peak at 923 cm^−1^ was associated with the oscillations of α-1,4-glycosidic bonds, and the band at 769 cm^−1^ was characteristic of C-C bond vibrations. According to previous reports, polysaccharides containing 1 → 4 glycosidic linkages typically exhibit characteristic absorption bands between 1175 and 1140 cm^−1^, which indicate the presence of glycosidic bonds [18].

The absorption band observed near 1047 cm^−1^ was attributed to ordered crystalline structures associated with starch helical arrangements, whereas the band at 1022 cm^−1^ corresponded to disordered amorphous regions. Therefore, the absorbance ratio at 1047 cm^−1^ 1022 cm^−1^ (R_1047/1022_) was calculated to assess the degree of short-range molecular order within the starch granules [18].

The 1047/1022 cm^−1^ values of all samples are summarized in Figure 3a. RB starch exhibited a relatively high R_1047/1022_ value, indicating a greater degree of short-range molecular order, whereas TB starch exhibited a relatively low value. The absorbance ratios of WQ, BK, OG, Ch, Mi, and RK starches showed minimal variation, suggesting that these starches possess comparable levels of short-range structural organization.

### 3.4. XRD

The crystalline structures of coarse-grain starches were analyzed using XRD. The classification of starch crystalline types was based on the presence or absence of characteristic diffraction peaks at 5.6°, 15.2°, 17.1°, 18.1°, and 23.1°, according to established criteria [24,25]. As shown in Figure 3b, all ten starch samples exhibited distinct diffraction peaks at 15.2°, 17.1°, and 23.1°. Starches from MB, TB, Mi, OG, and Ma also exhibited a weak diffraction peak at 18.1° and no peak at 5.6°, indicating an A-type crystalline pattern, consistent with previous reports [2,35]. Notably, MB starch exhibited an A-type crystalline pattern despite being derived from a legume, consistent with previous studies on mung bean starch [36]. This finding differs from the C-type pattern commonly observed in most other legume starches. In contrast, the remaining starches (RK, Ch, RB, BK, and WQ) exhibited a weaker diffraction peak at 5.6° in addition to the characteristic A-type peaks, indicating a C-type crystalline pattern [37].

The relative crystallinity values were 28.23%, 30.42%, 20.52%, 34.25%, 26.27%, 26.89%, 30.47%, 31.99%, 31.62%, and 21.33% for MB, RK, Mi, Ch, OG, TB, RB, BK, WQ, and Ma, respectively (Figure 3b). Among the analyzed starches, Ch exhibited the highest relative crystallinity (34.25%). In contrast, Mi starch exhibited the lowest relative crystallinity (20.52%), indicating significant differences in crystalline order among coarse-grain starches, which differed from those reported in some previous studies. Sarıkaya et al. [3] reported relative crystallinity values ranging between 21.2% and 27.4% for Virginia-grown kabuli chickpea starch, whereas Polesi et al. [38] reported a relative crystallinity of 42.05% for Brazilian chickpea starch (cultivar Cicero). The relative crystallinity of RB starch (30.47%) was comparable to the 32.70% crystallinity reported for red bean starch by Almeida et al. [39,40]. Qiao et al. [41] reported that starch crystallinity primarily arises from the ordered packing of amylopectin double helices, with amylose dispersed in amorphous regions, and consequently, lower amylose content generally correlates with higher crystallinity.

The differences in XRD patterns among coarse-grain starches were likely associated with variations in amylopectin content, unit cell structure, and microcrystalline arrangement [42]. Higher crystallinity, which is influenced by factors such as amylopectin chain length distribution, may enhance starch stability through stronger double-helix interactions and improved molecular orientation [43].

### 3.5. DSC

The gelatinization temperatures, including onset temperature (To), peak temperature (Tp), conclusion temperature (Tc), and gelatinization enthalpy (ΔH), of coarse-grain starches varied significantly among the different starch types. To, Tp, Tc, and ΔH ranged from 57.43 to 68.03 °C, 63.97 to 75.27 °C, 69.97 to 83.83 °C, and 5.06 to 12.70 J/g, respectively (Table 2).

Among the ten starches, RK starch exhibited the highest Tp (75.27 °C), Tc (83.83 °C), and ΔH (12.76 J/g), consistent with its high relative crystallinity (30.42%) and R_1047/1022_ ratio. These results indicate a more ordered crystalline structure and a greater energy requirement for gelatinization. In contrast, TB and RB starches exhibited lower ΔH values (5.59 and 5.06 J/g, respectively), corresponding to their lower crystallinity values (26.89% and 30.47%) and lower R_1047/1022_ ratios, suggesting less ordered molecular structures. WQ starch exhibited relatively low To (57.43 °C) and Tp (63.97 °C), which may be attributed to its exceptionally low amylose content (3.34%) and unique granule morphology, despite its moderate crystallinity (31.62%). These findings are consistent with previous observations that starch granules with higher crystallinity and greater short-range molecular order generally exhibit higher gelatinization temperatures and enthalpies [44].

The variations in gelatinization temperature and ΔH among starches from different coarse grains may result from differences in amylopectin chain length distribution [45], amylose content, crystal type, and granule morphology. ΔH represents the energy required to disrupt the double-helix structure of starch, which has been reported to correlate positively with the amylopectin content [46]. Longer amylopectin chains form extended double helices, which require higher temperatures for dissociation than shorter chains [47]. Kumar et al. [48] reported that the thermal properties of starch are correlated with the chain length distribution and average chain length of amylopectin molecules. Overall, the combined XRD, FTIR, and DSC results revealed a consistent trend: starches with higher crystallinity and greater short-range molecular order (e.g., RK) exhibited greater thermal stability, while those with lower structural order (e.g., TB and RB) required less energy for gelatinization.

Although Ch starch exhibited the highest relative crystallinity (34.25%) based on XRD analysis (Figure 3b), RK starch displayed the highest gelatinization enthalpy (ΔH = 12.76 J/g) and transition temperatures (Tp = 75.27 °C, Tc = 83.83 °C) (Table 2). This apparent discrepancy reflects the different structural characteristics evaluated by these techniques: XRD measures long-range crystalline order, while DSC ΔH reflects the energy required to disrupt double-helical structures. Therefore, RK starch may possess stronger double-helical interactions despite having slightly lower overall crystallinity than Ch starch.

### 3.6. Steady and Shear Measurements

**Figure 4:**
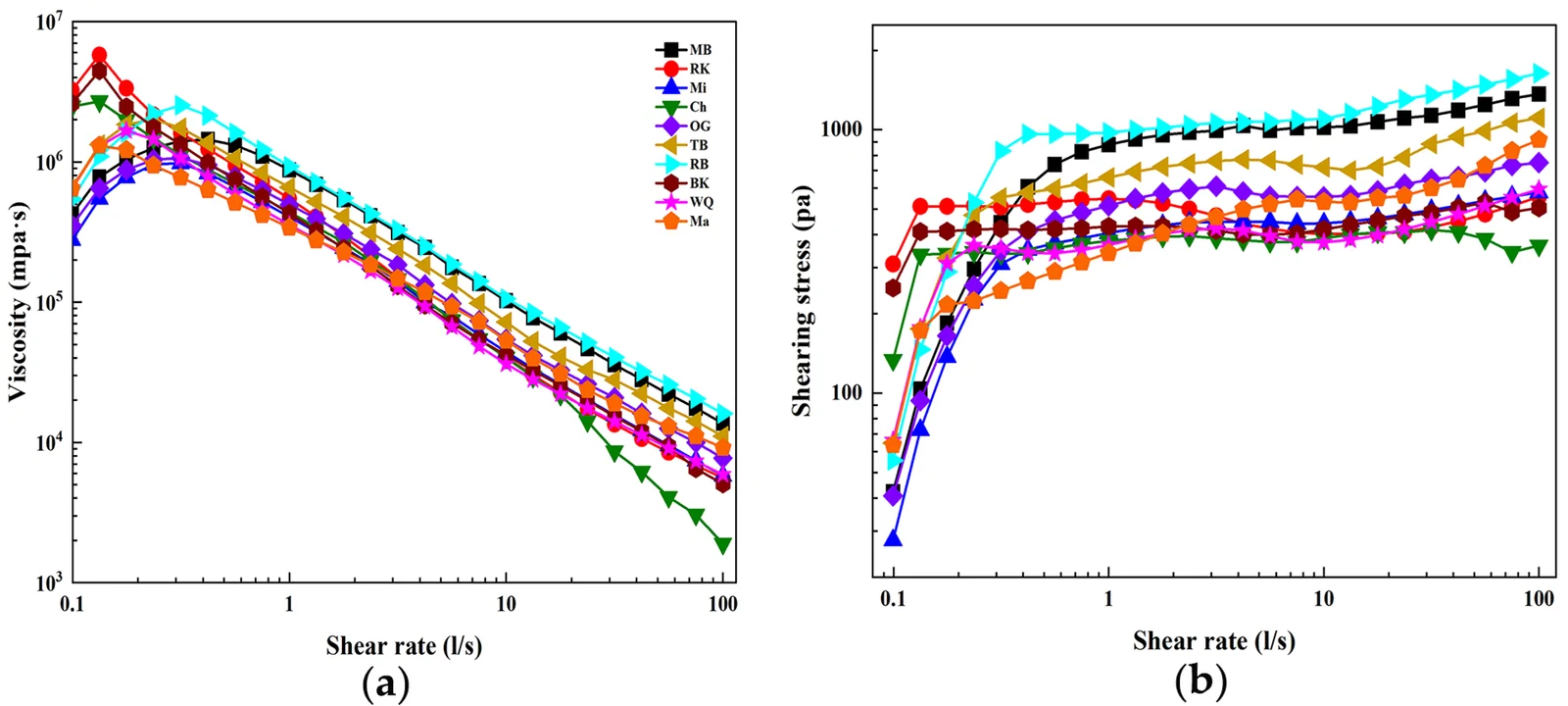
Rheological properties of the ten starches: (**a**) apparent viscosity as a function of shear rate and (**b**) shear stress as a function of shear rate.

Rheological properties are important indicators for evaluating the performance and stability of starches. They play a crucial role in determining the suitability of starches for various food industry applications [49]. Therefore, the apparent viscosity and viscoelastic properties of all the coarse-grain starches were investigated using shear rate sweep and frequency sweep tests. The variation in apparent viscosity with shear rate is shown in Figure 4. All starch samples exhibited typical non-Newtonian pseudoplastic behavior, characterized by shear-thinning as the shear rate increased from 0.1 to 100 s^−1^. Among the samples, RB, MB, and TB starches exhibited slightly higher apparent viscosities compared with the control Ma starch.

On the other hand, WQ, RK, BK, Mi, and Ch starches exhibited slightly lower apparent viscosities. The OG starch showed a viscosity pattern similar to that of Ma starch. The observed differences likely resulted from variations in the molecular structure and granule morphology of the starches.

The flow behavior of the experimental data was analyzed using the Herschel–Bulkley model. Most starch samples exhibited a high coefficient of determination (R^2^ > 0.90), indicating a good fit to the rheological characteristics of the starch pastes, except for MB starch (R^2^ = 0.872) and OG starch (R^2^ = 0.882). The relatively lower R^2^ values for MB and OG starches suggest that the Herschel–Bulkley model may not adequately describe the flow behavior of these two samples compared with the other starches. This deviation may be attributed to several factors. For MB starch, the exceptionally high peak viscosity (9278.00 cP) and consistency coefficient (K = 912.83) indicate the formation of a highly viscous paste that may exhibit more complex flow behavior, including time-dependent thixotropic effects that are not accounted for by the Herschel–Bulkley model. For OG starch, its relatively low trough viscosity (1359.33 cP) and unique granule morphology (oat starch typically exhibits irregular polygonal granules with a bimodal size distribution) may contribute to flow behavior that deviates from the ideal Herschel–Bulkley model. Despite these relatively lower R^2^ values, the model still provided a reasonable description of the shear-thinning behavior for all samples, as demonstrated by the good agreement between experimental data and model predictions (Figure 4a) and the consistent n values (all < 1), indicating pseudoplastic behavior.

The flow behavior index (n), consistency coefficient (K), and coefficient of determination (R^2^) are presented in Table 3 and were used to evaluate structural changes in starch structure under shear force. The consistency coefficient exhibited higher values for RB starch (928.21) and MB starch (912.83). The flow behavior index describes the relationship between shear stress and shear rate, indicating whether a sample exhibits shear-thinning or shear-thickening behavior. A value of n approaching zero or less than 1 indicates shear-thinning behavior. Similar trends were observed in the apparent viscosity-shear rate curves (Figure 4a), indicating that all starch samples exhibited shear-thinning behavior. The loss modulus, complex shear modulus, and dynamic viscosity were determined based on the stress–strain relationship described by Singh and Kaur [50]. As shown in Figure 4b, shear stress increased with increasing shear rate for all samples, demonstrating non-linear behavior and confirming that the starch paste exhibited non-Newtonian flow characteristics [51].

### 3.7. Dynamic Rheological Properties

**Figure 5:**
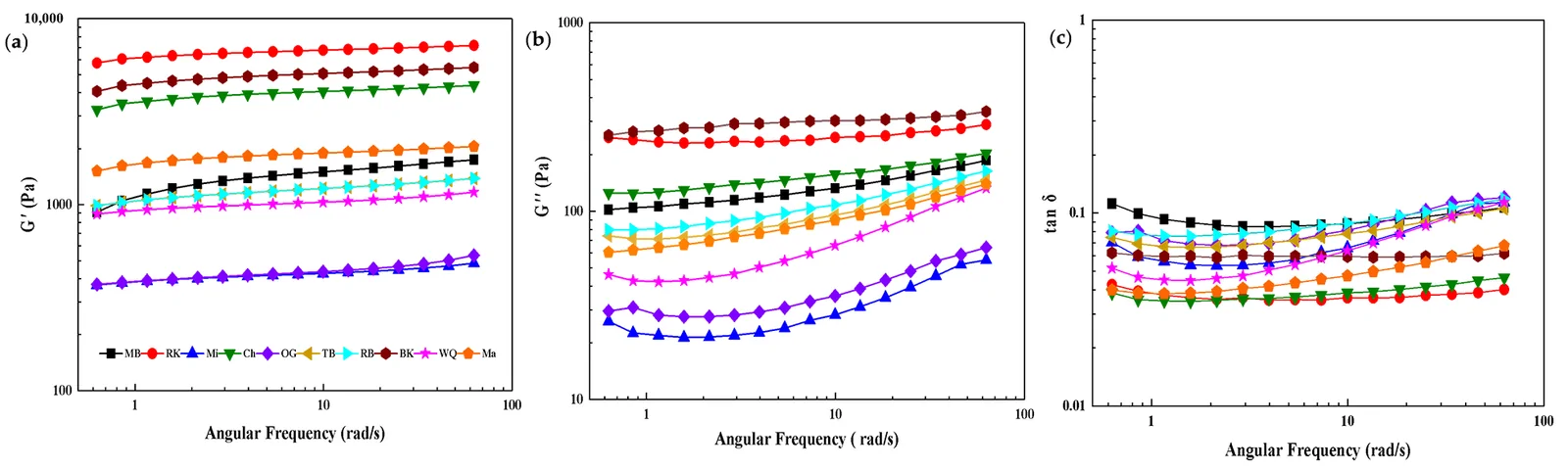
Rheological properties of the ten starches: (**a**) storage modulus (G′), (**b**) loss modulus (G″), and (**c**) loss tangent (tanδ).

As illustrated in Figure 5, both the storage modulus (G′) and the loss modulus (G′′) increased with frequency. G′ represents the elastic component, whereas G′′ represents the viscous component. Across the frequency range, the G′ values were higher than the G″ values for all starch pastes, indicating weak gel behavior characterized by more pronounced elastic properties than viscous properties. The viscoelastic behavior of starch pastes can be described by the loss tangent (tan δ), which represents the ratio of energy lost to energy stored per oscillation cycle [20]. As illustrated in Figure 5c, the loss factor (G″/G′) for all samples was below 1, indicating that elastic behavior predominated over viscous behavior. The values of G′, G″, and tan δ varied among the coarse-grain starches compared with the Ma starch gels. The elastic modulus of RK, BK, and Ch starch gels was higher than that of Ma and other coarse-grain starches, which exhibited comparable modulus patterns. The MB, RB, TB, and WQ starch gels exhibited G′, G″, and tan δ values comparable to those of Ma starch, indicating similar rheological behavior. In contrast, the OG and Mi starch gels exhibited lower G′ and G″ values than Ma gels, indicating a greater contribution of viscous behavior to their rheological response.

The observed variations in the rheological properties among the coarse-grain starch gels may be associated with differences in their structural and compositional characteristics. Notably, starches with higher amylose contents, such as RK (17.19%) and BK (20.63%), exhibited higher G′ values, consistent with previous reports that amylose plays an important role in promoting the formation of stronger gel networks [17]. Conversely, starches with lower amylose content, such as TB (14.21%) and RB (13.86%), displayed lower G′ values, suggesting weaker gel structures. Additionally, the differences in granule morphology (as observed by SEM, Section 3.2) and crystalline organization (as determined by XRD, Section 3.4) may also contribute to variations in rheological behavior. Although other factors, such as amylopectin branch-chain length and the presence of endogenous lipids or proteins, have been reported to affect starch gel rheology [43,52], these parameters were not directly measured in the present study. Therefore, these interpretations should be considered literature-supported possibilities rather than definitive conclusions, and future studies are needed to elucidate the contributions of these factors.

### 3.8. RVA

**Figure 6:**
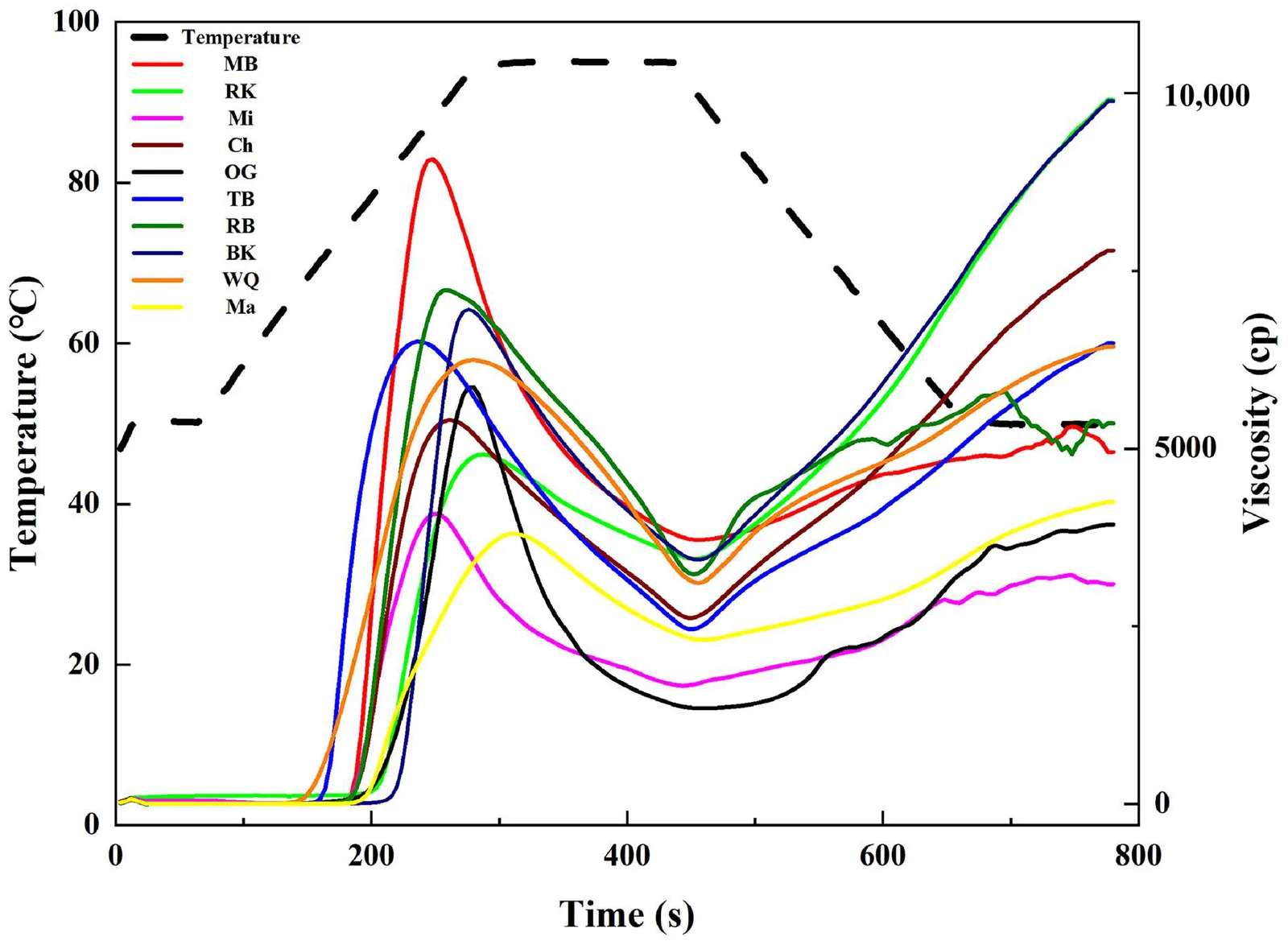
Viscometric profiles of starches from ten common coarse grains.

Significant differences in pasting properties were observed among the ten coarse-grain starch samples (Figure 6 and Table 4). Starch viscosity generally depends on its botanical source, granule size, crystalline structure, amylose content, and the amylose-to-amylopectin ratio [18]. As shown in Table 4, the peak viscosity of MB starch (9278.00 cP) was approximately twice that of Ma starch and significantly higher than that of other coarse-grain starches (*p* < 0.05). This observation indicates that MB starch has a higher water-absorption capacity [2], resulting in greater granule swelling and a more viscous paste, highlighting its potential as a food thickener. These findings were consistent with the report by Gunaratne et al. [36], which showed that mung bean starch with higher swelling capacity exhibited higher peak viscosity. Peak viscosity was closely related to the water absorption capacity and the degree of swelling of starch granules.

Trough viscosity is a critical parameter in starch processing because it reflects the ability of starch pastes to withstand heat and shear stress during processing. Starches with high trough viscosity are ideal for applications requiring consistent starch performance during prolonged cooking. In this study, MB starch exhibited the highest trough viscosity (3775.67 cP), while OG starch showed the lowest value (1359.33 cP). Compared with the control group (Ma starch), the trough viscosity of the remaining eight starches was significantly higher, except for OG starch (1343.00 cP). These results suggest that, for industrial applications involving prolonged cooking times, eight starches (excluding OG starch) may be more suitable than Ma starch.

Breakdown viscosity indicates the resistance of starch paste to heat and shear stress. Higher breakdown viscosity values correspond to lower stability of paste viscosity under heating and stirring. In this study, RK starch exhibited the lowest breakdown viscosity (1389.00 cP), which was not significantly different from that of Ma starch (1452.00 cP). Mi starch also showed relatively low breakdown viscosity values, suggesting comparable stability under thermal and shear stress. These findings indicate that RK and Mi starches exhibit superior resistance to heat and shear stress, with RK starch demonstrating the highest stability.

The setback viscosity represents the difference between the final viscosity and the trough viscosity, reflecting the extent of starch retrogradation and the tendency of the paste to undergo structural rearrangement during cooling. The setback viscosity values of RK (6587.67 cP), BK (6538.67 cP), and Ch (5799.00 cP) starches were approximately three times higher than those of Ma starch and significantly higher than those of the other coarse-grain starches (*p* < 0.05). Setback viscosity is closely associated with the retrogradation tendency and gelling ability of amylose [17]. The higher setback viscosities observed for RK, BK, and Ch starches indicated a greater tendency toward retrogradation compared with Ma starch.

Final viscosity reflects the stability of the starch paste upon cooling and the extent of polymerization of the starch molecules. It is measured at the end of cooling (50 °C) and indicates the ability of starch to form a stable paste or gel, as well as the stability of the cooked paste. In this study, the final viscosity of the ten coarse-grain starches ranged from 3091.50 cP (Mi starch) to 9950.33 cP (BK starch). Except for OG and Mi starches, the final viscosities of the remaining starches were significantly higher (*p* < 0.05) than that of Ma starch (4216.67 cP). These results suggest that eight of the ten starches, excluding OG and Mi, exhibit higher gel stability after cooling, making them suitable for applications requiring higher final viscosities.

The pasting temperatures of the ten common coarse-grain starches differed significantly (*p* < 0.05), with the highest value observed for BK starch (80.72 °C), followed by RK starch (78.53 °C), and the lowest in WQ starch (67.05 °C). The notably higher pasting temperatures of BK starch compared with the other starches warrant further discussion in relation to its thermal properties. As shown in Table 2, BK starch exhibited a DSC To of 63.47 °C and Tc of 83.83 °C, indicating that gelatinization occurred over a broad temperature range. The RVA pasting temperature of BK starch (80.72 °C) was near the upper limit of this range and approached the DSC conclusion temperature. This relatively high pasting temperature is consistent with the high relative crystallinity of BK starch (31.99%, Figure 3b) and its high short-range molecular order (R_1047/1022_ ratio), which may contribute to greater resistance to gelatinization. It is important to note that RVA pasting temperature reflects the onset of viscosity development during heating and is not directly equivalent to the DSC gelatinization To, because RVA measures viscosity changes under shear conditions, while DSC measures heat flow associated with the melting of crystalline regions. The high pasting temperature of BK starch suggests strong granular integrity, which is further supported by its low breakdown viscosity (3518.00 cP) and high final viscosity (9950.33 cP), indicating strong resistance to heat and shear stress.

The pasting temperatures of seven starches, excluding BK and RK, were lower than that of Ma starch (76.93 °C). Notably, WQ starch exhibited the lowest pasting temperature (67.05 °C), which may be attributed to its exceptionally low amylose content (3.34%) and small granule size, facilitating rapid water uptake, granule swelling, and gelatinization. Ma starch exhibited the highest peak time (5.20 min), while TB starch exhibited the lowest (3.93 min). The peak times of all coarse-grain starches were lower than that of maize starch.

The pasting properties of starch are influenced by multiple factors, including amylose content, amylopectin structure, granule size, crystalline structure, and molecular order [17,18]. The combined XRD, DSC, and RVA results from this study reveal consistent trends: starches with higher crystallinity and greater short-range molecular order (e.g., BK and RK) generally exhibited higher pasting temperatures and greater resistance to thermal and shear stress, while starches with lower structural order and lower amylose content (e.g., WQ) exhibited lower pasting temperatures and more rapid gelatinization.

### 3.9. Gel Texture

In many food products, starch molecules form a three-dimensional gel network through junction zones stabilized by hydrogen bonds, hydrophobic interactions (van der Waals forces), ionic cross-bridges, molecular entanglements, and covalent bonds [42]. As a result, starch gels exhibit both solid- and liquid-like properties, indicating their viscoelastic nature. Gel strength is affected by multiple factors, including the structure and properties of starch granules, starch composition, molecular interactions with water, and the conditions under which the gel forms [42,53].

The texture properties of starch gels were further evaluated using TPA (Table 5). TPA parameters, including hardness, cohesiveness, springiness, stickiness (defined as the maximum negative force required to separate the probe from the sample surface during the withdrawal phase of the first compression cycle), chewiness, and adhesiveness, differed significantly among starch gels derived from different coarse-grains.

RK starch gels exhibited the highest hardness (12.66 ± 1.11 N), cohesiveness (3.48 ± 0.07), springiness (7.05 ± 0.54 mm), stickiness (28.22 ± 1.55 N), adhesiveness (6.35 ± 0.02 N), and chewiness (24.37 ± 1.71 mJ), indicating superior gel texture properties. This observation is consistent with the rheological results, where RK starch also exhibited the higher storage modulus (G′) among all samples (Figure 5a), confirming its superior gel-forming ability. In contrast, OG starch gels showed the lowest hardness (0.29 ± 0.01 N). These results suggest that RK starch is particularly suitable for producing smooth and chewy food products.

Gel strength is primarily influenced by the extent of the amylose gel network and the deformability of swollen starch granules. Furthermore, apparent amylose content was positively correlated with gel hardness [17], with starches that form firmer gels generally containing higher amylose content and longer amylopectin chains.

### 3.10. In Vitro Digestibility

Starch digestibility is an important indicator of the nutritional quality of both legume and cereal starches, reflecting the potential health benefits associated with these underutilized crops. It is influenced by multiple structural and compositional factors, including the botanical origin of the starch, granule size distribution, amylose-to-amylopectin ratio, amylopectin branch chain length, crystallinity patterns, polymorphic forms, and surface morphological features such as pores, fissures, and channels [17].

**Figure 7:**
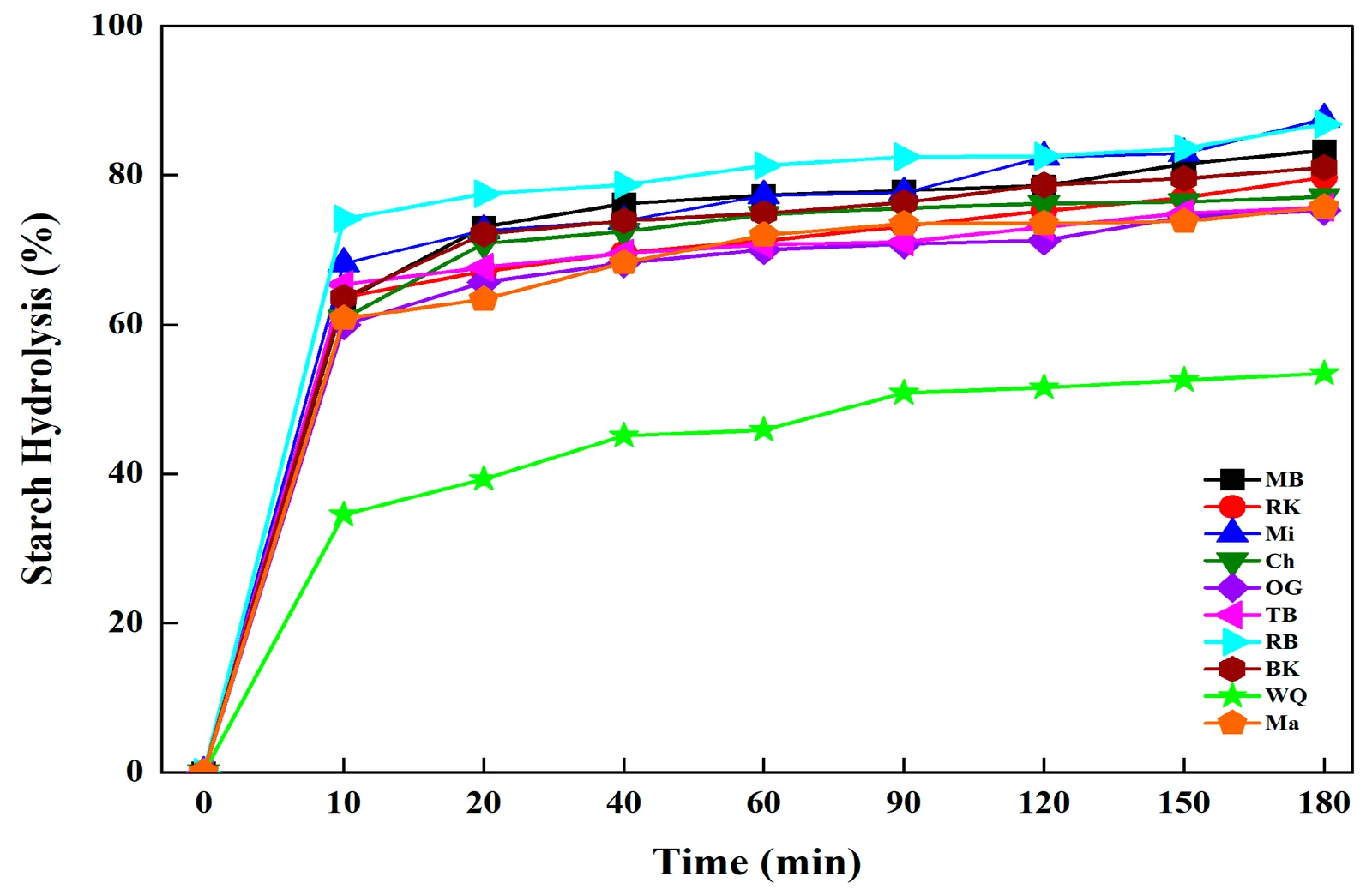
In vitro digestograms of starches from ten common coarse grains.

Comprehensive analysis of ten common coarse-grain starch varieties revealed significant differences (*p* < 0.05) in the proportions of RDS, SDS, and RS (Table 6 and Figure 7). RB starch exhibited the highest RDS content (78.00 ± 0.12%), whereas WQ starch had the highest SDS (12.42 ± 0.49%) and RS (47.83 ± 2.44%). Conversely, WQ starch showed the lowest RDS (39.76 ± 1.95%), RB starch had the lowest SDS (5.03 ± 0.42%), and MB starch contained the least RS (16.97 ± 0.54%).

The elevated RS content of WQ starch indicates strong resistance to in vitro digestion, which has been associated with potential health benefits, such as improved glycemic control and reduced colorectal cancer, in previous reports [54].

The exceptionally high RS content (47.83%) and low equilibrium hydrolysis percentage (C_∞_ = 53.42%) of WQ starch warrant further mechanistic interpretation in relation to its structural features. First, WQ starch exhibited a C-type crystalline pattern, as determined by XRD (Section 3.4), which is generally considered more resistant to enzymatic hydrolysis than A-type starches due to its intermediate crystalline organization [52]. Second, WQ starch exhibited an exceptionally low amylose content (3.34%) and relatively small granule size, as observed by SEM (Section 3.2), which may limit the accessibility of digestive enzymes to the starch granule surface. Third, the high short-range molecular order reflected by its R_1047_/_1022_ ratio (Table 1) suggests a compact granular structure that may restrict enzyme penetration. The combination of these structural characteristics, including C-type crystallinity, small granule size, and high short-range molecular order, likely contributes to the high RS content and strong resistance to digestion of WQ starch. These findings highlight white quinoa starch as a promising ingredient for developing low-glycemic-index functional foods.

The equilibrium digestion percentages (C_∞_) differed significantly among the starch samples (*p* < 0.05), with values ranging from 53.42 ± 3.48% to 87.52 ± 3.53% (Table 6). Mi starch (87.52 ± 3.53%) and RB starch (86.8 ± 2.52%) exhibited near-complete digestibility (high C_∞_), while WQ starch demonstrated exceptional enzymatic resistance, with the lowest C_∞_ value (53.42 ± 3.48%).

The digestion kinetic parameters (K values) differed significantly, ranging from 4.64 to 7.08 × 10^−2^ min^−1^, with Ch starch demonstrating the most rapid hydrolysis (7.08 ± 0.48 × 10^−2^ min^−1^), exceeding the rate observed for WQ starch (5.40 ± 0.3 × 10^−2^ min^−1^).

This study systematically characterized the digestibility profiles of ten common coarse-grain starches, revealing wide variations in RDS (39.76 to 78.00%), SDS (5.03 to 12.42%), and RS (16.97 to 47.83%) contents among the starch varieties. The exceptionally high RS content (47.83%) and low equilibrium hydrolysis percentage (C_∞_ = 53.42%) of WQ starch, together with its distinct structural characteristics identified by XRD, FTIR, and SEM, highlight its potential as a promising ingredient for developing low-glycemic-index functional foods. These findings provide a scientific basis for the targeted selection of coarse-grain starches in the formulation of starch-based foods with controlled digestibility to meet specific dietary and health objectives.

### 3.11. Correlation Analysis

**Figure 8:**
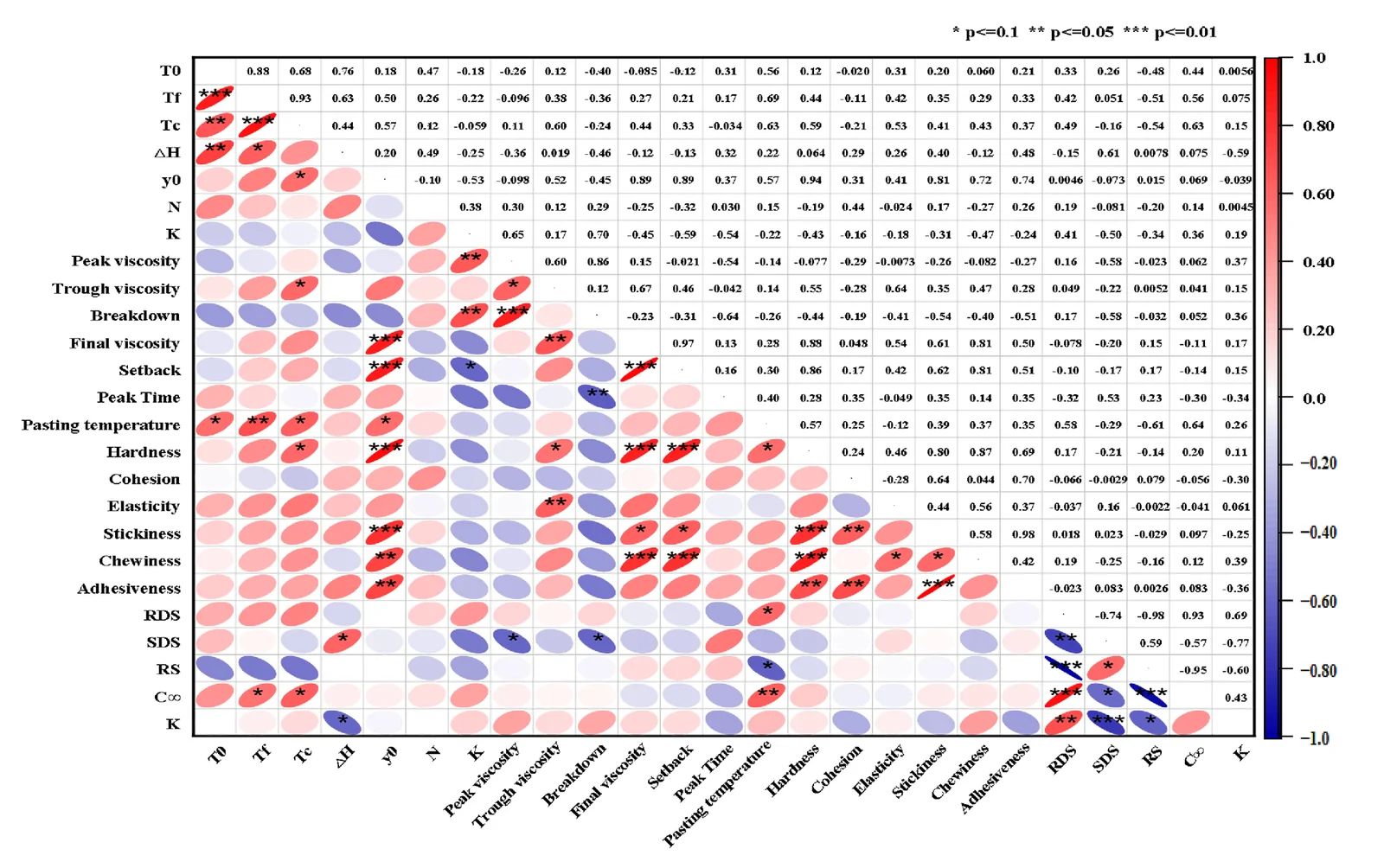
Correlation analysis of key quality properties of starches from ten common coarse grains (* *p* ≤ 0.10, ** *p* ≤ 0.05, *** *p* ≤ 0.01; red lines indicate positive correlations, and blue lines indicate negative correlations).

Pearson correlation analysis of the physicochemical, pasting, rheological, textural, and digestive properties of different starches is presented in Figure 8. Gelatinization enthalpy (ΔH) showed highly significant positive correlations with the gelatinization characteristic temperatures (To, Tp, and Tc; *p* < 0.01), consistent with the thermodynamic principles of starch gelatinization. This result indicates that starches with a higher degree of crystalline order require greater energy to disrupt double-helical structures during thermal gelatinization, thereby resulting in higher gelatinization temperatures. Moreover, significant correlations were observed between digestive fractions (RDS, SDS, and RS) and multiple pasting and textural parameters, confirming that starch pasting and textural properties are significantly associated with starch digestibility. These statistical results further substantiate the structural-function relationships underlying the differences in digestibility among the ten coarse-grain starches. Furthermore, the observed correlation patterns are consistent with previous reports on cereal and legume starches, further supporting the important contribution of pasting-texture interactions in determining starch digestive behavior [48,55].

## 4. Conclusions

This study systematically evaluated the structural, functional, and digestibility characteristics of ten coarse-grain starches, revealing distinct differences associated with their botanical origins. Among the investigated starches, red kidney bean starch exhibited the highest gel strength and viscosity, which were attributed to its high amylose content and crystalline order. These characteristics suggest its potential as a natural thickening or gelling agent in food formulations. White quinoa starch exhibited exceptional resistance to enzymatic digestion (RS content: 47.83%), which is associated with its C-type crystalline pattern and low amylose content, highlighting its potential as a functional ingredient for low-glycemic-index foods. Chickpea starch exhibited the highest relative crystallinity (34.25%) and enhanced thermal stability, suggesting its suitability for high-temperature food processing applications. The combined XRD, FTIR, and DSC analyses demonstrated that starch functionality is governed by the synergistic effects of long-range crystalline order, short-range molecular order, and granule morphology rather than by any single structural characteristic.

These findings highlight the potential for targeted selection of starches for specific applications, including enzyme-resistant starches for low-glycemic-index foods, high-viscosity starches for texture modification, and thermally stable starches for high-temperature processing. Overall, this study provides a comprehensive understanding of the structure–function relationships of coarse-grain starches and offers a theoretical basis for developing functional foods with tailored starch properties, including low-glycemic-index products and specialized complementary foods. However, further validation is required before practical applications can be established. Future research could explore molecular and composite modification strategies to improve processing performance and nutritional functionality. Additionally, molecular weight distribution and amylopectin chain length distribution were not determined in this study. Further characterization of these structural characteristics is needed to elucidate the structure–function relationships of coarse-grain starches.

A limitation of this study is that the coarse-grain samples were obtained from a commercial market without documented cultivar information or harvest year, which may affect the reproducibility and generalizability of the results. Future studies should incorporate authenticated cultivars grown under controlled conditions to validate the structure–function relationships identified in this study.

Additionally, only one batch of each grain sample was used for starch isolation because the samples were commercially obtained from a single local market. Therefore, the characteristics observed for each grain may not fully represent the broader variability within the corresponding species or variety. Future studies should evaluate multiple batches or authenticated cultivars obtained from different sources to confirm the robustness and generalizability of these findings.

## Figures and Tables

**Table 1:** Amylose content, moisture content, ash content, and color parameters of starches from ten common coarse grains.

Starch Sample	L*	a*	b*	ΔE	W	Moisture (%)	Ash (g/100 g)	Amylose (%)
MB	95.69 ± 0.03 ^e^	−1.84 ± 0.01 ^k^	5.36 ± 0.02 ^d^	2.51 ± 0.02 ^g^	90.41 ± 0.05 ^h^	6.59 ± 0.10 ^f^	0.04 ± 0.01 ^d^	20.89 ± 0.74 ^ab^
RK	94.43 ± 0.02 ^h^	0.06 ± 0.02 ^g^	3.39 ± 0.03 ^f^	0.71 ± 0.02 ^j^	96.59 ± 0.02 ^e^	5.87 ± 0.03 ^g^	0.03 ± 0.00 ^d^	17.19 ± 0.44 ^c^
Mi	95.62 ± 0.03 ^f^	−0.16 ± 0.01 ^i^	2.77 ± 0.02 ^g^	1.81 ± 0.03 ^h^	97.15 ± 0.02 ^d^	7.88 ± 0.08 ^d^	0.28 ± 0.01 ^b^	15.31 ± 1.00 ^d^
Ch	95.25 ± 0.01 ^g^	0.02 ± 0.00 ^h^	2.73 ± 0.00 ^g^	1.61 ± 0.01 ^i^	97.27 ± 0.00 ^d^	9.15 ± 0.33 ^b^	0.05 ± 0.02 ^cd^	19.65 ± 1.20 ^b^
OG	96.15 ± 0.03 ^c^	0.16 ± 0.02 ^f^	1.43 ± 0.01 ^i^	3.18 ± 0.01 ^e^	98.45 ± 0.03 ^a^	7.29 ± 0.02 ^e^	0.09 ± 0.00 ^cd^	21.81 ± 1.09 ^a^
TB	95.88 ± 0.01 ^d^	0.35 ± 0.01 ^d^	2.06 ± 0.01 ^h^	2.58 ± 0.02 ^g^	97.47 ± 0.02 ^c^	6.57 ± 0.21 ^f^	0.08 ± 0.09 ^cd^	14.21 ± 0.86 ^de^
RB	88.75 ± 0.04 ^j^	1.00 ± 0.01 ^c^	11.11 ± 0.07 ^a^	9.16 ± 0.05 ^a^	84.16 ± 0.05 ^i^	6.02 ± 0.34 ^g^	0.13 ± 0.08 ^c^	13.86 ± 0.68 ^e^
BK	91.73 ± 0.04 ^i^	−1.84 ± 0.01 ^f^	5.36 ± 0.01 ^c^	2.51 ± 0.03 ^e^	90.41 ± 0.02 ^f^	7.10 ± 0.03 ^e^	0.08 ± 0.10 ^cd^	20.63 ± 1.83 ^ab^
WQ	96.34 ± 0.04 ^b^	0.25 ± 0.01 ^e^	2.03 ± 0.08 ^h^	2.89 ± 0.06 ^f^	97.77 ± 0.08 ^b^	8.37 ± 0.53 ^c^	0.40 ± 0.14 ^a^	3.34 ± 0.17 ^f^
Ma	96.57 ± 0.02 ^a^	−1.19 ± 0.02 ^g^	6.74 ± 0.05 ^b^	3.76 ± 0.05 ^c^	92.13 ± 0.06 ^g^	11.47 ± 0.01 ^a^	0.08 ± 0.01 ^cd^	19.68 ± 0.08 ^b^

Note: Amylose content is expressed as a percentage of total starch (%, starch basis). Moisture and ash contents are expressed as percentages of sample weight on an as-is basis. Data are presented as mean ± standard deviation (*n* = 3). Different superscript letters within the same column indicate significant differences (*p* < 0.05) according to Duncan’s multiple range test.

**Table 2:** Thermal Properties of starches from ten common coarse grains.

Starch Sample	To/°C	Tp/°C	Tc/°C	△H/(J/g)
MB	66.58 ± 0.40 ^b^	73.60 ± 1.33 ^abc^	81.75 ± 2.79 ^b^	11.04 ± 1.35 ^b^
RK	64.62 ± 0.71 ^cd^	74.04 ± 0.26 ^ab^	82.72 ± 0.53 ^a^	12.76 ± 0.49 ^a^
Mi	65.88 ± 0.85 ^b^	73.20 ± 1.83 ^bc^	80.48 ± 1.75 ^c^	12.70 ± 2.89 ^a^
Ch	60.80 ± 0.26 ^e^	68.73 ± 0.25 ^d^	78.10 ± 1.35 ^d^	5.73 ± 1.74 ^e^
OG	58.37 ± 0.55 ^f^	63.67 ± 0.32 ^f^	69.97 ± 0.67 ^g^	6.77 ± 1.47 ^f^
TB	58.30 ± 0.17 ^f^	66.57 ± 0.12 ^f^	75.07 ± 0.25 ^e^	5.59 ± 0.83 ^g^
RB	57.87 ± 0.4 ^fg^	66.80 ± 0.96 ^e^	78.07 ± 0.70 ^d^	5.06 ± 0.44 ^g^
BK	63.47 ± 0.57 ^d^	75.27 ± 1.20 ^a^	83.83 ± 0.64 ^a^	7.90 ± 0.96 ^e^
WQ	57.43 ± 0.21 ^g^	63.97 ± 0.06 ^f^	71.97 ± 0.64 ^f^	9.31 ± 1.97 ^d^
Ma	68.03 ± 0.61 ^a^	72.70 ± 0.17 ^c^	77.50 ± 0.95 ^d^	10.51 ± 0.88 ^c^

Different superscript letters within the same column indicate significant differences (*p* < 0.05).

**Table 3:** Steady-state rheological parameters of starches from ten common coarse grains based on the Herschel–Bulkley Model.

Starch Sample	σ_0_	n	K/pa.s^n^	R^2^
MB	42.288	0.864	912.83	0.872
RK	309.198	0.466	263.55	0.970
Mi	27.660	0.198	373.64	0.940
Ch	133.757	0.112	240.08	0.921
OG	40.760	0.449	488.11	0.882
TB	64.638	0.199	589.46	0.952
RB	55.510	0.059	928.21	0.962
BK	250.813	0.065	175.58	0.944
WQ	66.188	0.150	303.34	0.964
Ma	63.584	0.346	273.46	0.997

Note: σ_0_ = yield stress; n = flow behavior index; K = consistency coefficient; R = coefficient of determination.

**Table 4:** Pasting properties of starches from ten common coarse grains.

Starch Sample	Peak Viscosity (cP)	Trough Viscosity (cP)	Breakdown (cP)	Final Viscosity (cP)	Setback (cP)	Peak Time (min)	Pasting Temperature (°C)
MB	9278.00 ± 40.95 ^a^	3775.67 ± 4.73 ^a^	5502.33 ± 37.54 ^a^	4998.00 ± 795.90 ^de^	1549.00 ± 228.63 ^g^	4.11 ± 0.03 ^e^	74.87 ± 0.49 ^e^
RK	4849.00 ± 155.02 ^h^	3460.00 ± 164.70 ^b^	1389.00 ± 57.51 ^h^	9916.00 ± 490.08 ^a^	6587.67 ± 137.04 ^a^	4.85 ± 0.04 ^b^	78.53 ± 0.49 ^b^
Mi	4154.33 ± 118.50 ^i^	1636.00 ± 114.00 ^f^	2518.33 ± 232.50 ^f^	3091.33 ± 228.50 ^h^	1455.33 ± 114.50 ^g^	4.14 ± 0.14 ^e^	74.68 ± 0.42 ^e^
CH	6118.33 ± 73.57 ^f^	3081.00 ± 58.92 ^c^	3037.33 ± 128.19 ^e^	8880.00 ± 122.17 ^b^	5799.00 ± 96.63 ^b^	4.29 ± 0.03 ^d^	74.27 ± 0.03 ^e^
OG	5864.33 ± 91.13 ^g^	1359.33 ± 46.18 ^g^	4505.00 ± 77.54 ^b^	3809.33 ± 142.90 ^g^	2450.00 ± 175.82 ^e^	4.62 ± 0.04 ^c^	76.12 ± 0.51 ^d^
TB	6503.33 ± 84.50 ^d^	2455.00 ± 85.00 ^e^	4048.33 ± 169.50 ^c^	6483.00 ± 48.00 ^c^	4028.00 ± 37.00 ^c^	3.93 ± 0.00 ^f^	70.18 ± 0.02 ^g^
RB	7231.33 ± 9.50 ^b^	3231.00 ± 130.00 ^c^	4000.33 ± 139.5 ^c^	5354.33 ± 734.50 ^d^	2123.33 ± 604.5 ^f^	4.30 ± 0.03 ^d^	74.65 ± 0.40 ^e^
BK	7021.00 ± 94.30 ^c^	3503.00 ± 177.55 ^b^	3518.00 ± 185.52 ^d^	9950.33 ± 197.76 ^a^	6538.67 ± 66.71 ^a^	4.60 ± 0.07 ^c^	80.72 ± 0.78 ^a^
WQ	6319.00 ± 204.71 ^e^	2956.33 ± 133.42 ^d^	3362.67 ± 324.6 ^d^	6465.00 ± 101.89 ^c^	3508.67 ± 211.41 ^d^	4.62 ± 0.10 ^c^	67.05 ± 0.05 ^h^
Ma	3756.67 ± 54.56 ^j^	2304.67 ± 42.03 ^e^	1452.00 ± 47.95 ^h^	4216.67 ± 35.53 ^fg^	1912.00 ± 69.40 ^f^	5.20 ± 0.07 ^a^	76.93 ± 0.49 ^c^

Different superscript letters within the same column indicate significant differences (*p* < 0.05).

**Table 5:** Texture profile analysis of starch gels from ten common coarse grains.

Starch Sample	Hardness (N)	Cohesiveness (ratio)	Springiness (mm)	Stickiness (N)	Chewiness (mJ)	Adhesiveness (N)
MB	0.46 ± 0.02 ^d^	0.89 ± 0.02 ^a^	6.18 ± 0.29 ^b^	0.41 ± 0.01 ^d^	2.51 ± 0.17 ^d^	0.14 ± 0.01 ^abc^
RK	12.66 ± 1.11 ^a^	3.48 ± 0.07 ^b^	7.05 ± 0.54 ^ab^	28.22 ± 1.55 ^a^	24.37 ± 1.71 ^a^	6.35 ± 0.02 ^c^
Mi	0.40 ± 0.16 ^d^	0.9 ± 0.03 ^a^	5.46 ± 2.02 ^bc^	0.36 ± 0.14 ^d^	2.16 ± 1.14 ^d^	0.14 ± 0.01 ^abc^
Ch	7.87 ± 0.71 ^b^	0.63 ± 0.01 ^b^	6.98 ± 0.15 ^a^	4.92 ± 0.45 ^b^	34.37 ± 3.61 ^b^	0.03 ± 0.00 ^c^
OG	0.29 ± 0.01 ^d^	3.28 ± 0.02 ^a^	2.96 ± 0.38 ^de^	0.85 ± 0.01 ^d^	0.81 ± 0.16 ^d^	0.24 ± 0.01 ^ab^
TB	0.67 ± 0.43 ^d^	0.89 ± 0.01 ^a^	6.21 ± 0.50 ^d^	0.6 ± 0.38 ^d^	3.86 ± 2.65 ^d^	0.12 ± 0.06 ^ab^
RB	3.94 ± 0.29 ^c^	0.38 ± 0.04 ^c^	5.35 ± 0.48 ^f^	1.51 ± 0.12 ^cd^	8.07 ± 0.73 ^cd^	0.07 ± 0.04 ^bc^
BK	8.94 ± 0.76 ^b^	0.35 ± 0.14 ^c^	5.44 ± 0.34 ^ef^	3.19 ± 1.35 ^c^	17.58 ± 7.60 ^c^	0.19 ± 0.16 ^a^
WQ	0.61 ± 0.03 ^d^	0.86 ± 0.03 ^a^	6.01 ± 0.08 ^cd^	0.52 ± 0.04 ^d^	3.14 ± 0.27 ^d^	0.09 ± 0.01 ^bc^
Ma	1.15 ± 0.04 ^d^	0.85 ± 0.02 ^a^	6.01 ± 0.43 ^de^	0.98 ± 0.06 ^d^	5.88 ± 0.35 ^d^	0.06 ± 0.01 ^c^

Different superscript letters within the same column indicate significant differences (*p* < 0.05).

**Table 6:** In vitro digestibility of starches from ten common coarse grains.

Starch Sample	RDS (%)	SDS (%)	RS (%)	C_∞_ (%)	K (10^−2^.min^−1^)
MB	76.58 ± 1.68 ^a^	5.80 ± 2.06 ^a^	17.62 ± 0.38 ^cd^	83.29 ± 1.89 ^ab^	6.30 ± 0.81 ^ab^
RK	68.00 ± 2.07 ^d^	8.25 ± 1.08 ^a^	23.74 ± 0.99 ^bc^	79.7 ± 4.51 ^ab^	5.18 ± 0.14 ^ab^
Mi	72.60 ± 0.94 ^bc^	9.82 ± 0.92 ^a^	17.58 ± 0.02 ^d^	87.52 ± 3.53 ^a^	5.29 ± 0.24 ^ab^
Ch	74.10 ± 1.95 ^ab^	5.56 ± 1.67 ^a^	20.34 ± 3.63 ^bcd^	77.11 ± 3.35 ^b^	7.08 ± 0.48 ^a^
OG	69.06 ± 0.3 ^cd^	5.91 ± 0.93 ^a^	25.03 ± 1.23 ^b^	75.23 ± 1.79 ^b^	5.96 ± 0.34 ^ab^
TB	70.39 ± 0.34 ^bcd^	5.64 ± 1.39 ^a^	23.97 ± 1.73 ^b^	75.68 ± 1.14 ^b^	6.33 ± 0.57 ^ab^
RB	78.00 ± 0.12 ^a^	5.03 ± 0.42 ^a^	16.97 ± 0.54 ^d^	86.8 ± 2.52 ^a^	5.99 ± 0.47 ^ab^
BK	70.41 ± 1.31 ^bcd^	6.43 ± 1.84 ^a^	23.16 ± 0.53 ^bc^	81.01 ± 0.1 ^ab^	6.20 ± 0.80 ^ab^
WQ	39.76 ± 1.95 ^e^	12.42 ± 0.49 ^a^	47.83 ± 2.44 ^a^	53.42 ± 3.48 ^c^	4.64 ± 0.03 ^b^
Ma	68.55 ± 0.63 ^d^	10.97 ± 4.79 ^a^	20.48 ± 4.16 ^bcd^	75.68 ± 1.34 ^b^	5.89 ± 0.60 ^ab^

Different superscript letters within the same column indicate significant differences (*p* < 0.05).

## Data Availability

The original contributions presented in this study are included in the article. Further inquiries can be directed to the corresponding authors.
